# Oligodendrocyte development in the embryonic tuberal hypothalamus and the influence of Ascl1

**DOI:** 10.1186/s13064-016-0075-9

**Published:** 2016-11-18

**Authors:** Candace M. Marsters, Jessica M. Rosin, Hayley F. Thornton, Shaghayegh Aslanpour, Natasha Klenin, Grey Wilkinson, Carol Schuurmans, Quentin J. Pittman, Deborah M. Kurrasch

**Affiliations:** 1 Department of Medical Genetics, Cumming School of Medicine, University of Calgary, Calgary, AB T2N 4N1 Canada; 2 Department of Biochemistry and Molecular Biology, Cumming School of Medicine, University of Calgary, Calgary, AB T2N 4N1 Canada; 3 Department of Pharmacology & Physiology, Cumming School of Medicine, University of Calgary, Calgary, AB T2N 4N1 Canada; 4 Hotchkiss Brain Institute, University of Calgary, Calgary, AB T2N 4N1 Canada; 5 Alberta Children’s Hospital Research Institute, University of Calgary, Calgary, AB T2N 4N1 Canada; 6 Biological Sciences Platform, Sunnybrook Research Institute, Toronto, ON M4N 3M5 Canada

**Keywords:** Gliogenesis, Oligodendrogenesis, Astrocyte, Ascl1, Neurog2, Sox9, Olig2, PdgfRα

## Abstract

**Background:**

Although the vast majority of cells in our brains are glia, we are only beginning to understand programs governing their development, especially within the embryonic hypothalamus. In mice, gliogenesis is a protracted process that begins during embryonic stages and continues into the early postnatal period, with glial progenitors first producing oligodendrocyte precursor cells, which then differentiate into pro-oligodendrocytes, pro-myelinating oligodendrocytes, and finally, mature myelinating oligodendrocytes. The exact timing of the transition from neurogenesis to gliogenesis and the subsequent differentiation of glial lineages remains unknown for most of the Central Nervous System (CNS), and is especially true for the hypothalamus.

**Methods:**

Here we used mouse embryonic brain samples to determine the onset of gliogenesis and expansion of glial populations in the tuberal hypothalamus using glial markers Sox9, Sox10, Olig2, PdgfRα, Aldh1L1, and MBP. We further employed *Ascl1* and *Neurog2* mutant mice to probe the influence of these proneual genes on developing embryonic gliogenic populations.

**Results:**

Using marker analyses for glial precursors, we found that gliogenesis commences just prior to E13.5 in the tuberal hypothalamus, beginning with the detection of glioblast and oligodendrocyte precursor cell markers in a restricted domain adjacent to the third ventricle. Sox9+ and Olig2+ glioblasts are also observed in the mantle region from E13.5 onwards, many of which are Ki67+ proliferating cells, and peaks at E17.5. Using *Ascl1* and *Neurog2* mutant mice to investigate the influence of these bHLH transcription factors on the progression of gliogenesis in the tuberal hypothalamus, we found that the elimination of *Ascl1* resulted in an increase in oligodendrocyte cells throughout the expansive period of oligodendrogenesis.

**Conclusion:**

Our results are the first to define the timing of gliogenesis in the tuberal hypothalamus and indicate that Ascl1 is required to repress oligodendrocyte differentiation within this brain region.

**Electronic supplementary material:**

The online version of this article (doi:10.1186/s13064-016-0075-9) contains supplementary material, which is available to authorized users.

## Background

The tuberal hypothalamus, consisting of the ventromedial hypothalamus (VMH), dorsomedial hypothalamus (DMH), and arcuate nucleus (ARC), is a key regulator of many important biological functions, such as energy balance, sexual behavior, thermoregulation, and affective functioning [1–4]. Although most of the research within this brain region is focused on understanding neuronal differentiation and function, the glial cells that interact with hypothalamic neurons also play a critical role in controlling homeostatic mechanisms, particularly aspects of feeding regulation [5, 6].

Gliogenesis is the developmental process of generating the supportive and active signalling central nervous system (CNS) glial cells, namely oligodendrocytes and astrocytes. Temporally, gliogenesis has been shown to follow embryonic neurogenesis in the CNS. Indeed, the differentiation of oligodendrocytes, which are the last cell type to differentiate in the CNS, has been well defined in the cortex and spinal cord and begins around embryonic day (E) 12.5, occurring in three consecutive waves each from its own distinct domain, as has been extensively reviewed elsewhere [7, 8]. Briefly, oligodendrocytes arise from Sox9+ progenitor cells that express the transcription factor Olig2 within restricted regions of the ventral neuroepithelium throughout the rostrocaudal axis. These progenitors further mature and start to migrate outwards after starting to express Sox10 and PdgfRα. Once in the mantle region, these glioblasts begin to actively proliferate and give rise to the many oligodendrocyte precursor cells (OPCs) that are necessary to adequately populate the mantle region. Temporally, these OPCs continue to mature and upon reaching their final maturation, these oligodendrocytes begin to myelinate axons [9–12]. Within the tuberal hypothalamus, neural progenitors that line the third ventricle are known to give rise to both neurons and glia [13]. Yet, despite considerable understanding of glial development in other brain regions, primarily the cortex and spinal cord, the timing for gliogenesis in the hypothalamus remains undefined.

Interestingly, genes of the basic helix-loop-helix family (bHLH), particularly *Olig2* and proneural genes *Neurogenin 2* (*Neurog2*) and *Achaete*-*scute homolog1* (*Ascl1*) also influence gliogenesis [14]. For example, during neurogenesis Olig2 has well-defined roles in the development of motor neurons in the spinal cord [15] and GABAergic neurons in the cortex [16, 17], while later it is required for the development of oligodendrocytes in both brain regions and, to a much lesser extent, ventrally-located astrocytes as observed in the forebrain and spinal cord [15, 18, 19].

In the case of proneural genes, their function during gliogenesis is more varied. For instance, ectopic expression of *Ascl1* in the cerebellum has been shown to increase the numbers of interneurons while concomitantly supressing an astrocytic fate; the loss of *Ascl1* exhibits the opposite phenotype [16], suggesting that *Ascl1* restricts the differentiation of a shared progenitor pool into astrocytic lineages. Similarly in the cortex, *Neurog2* and *Ascl1* double knockout animals show increases in an astrocytic fate at the expense of neurons [20], while a single *Ascl1* knockout shows defects in populations of early-born Pdgfrα+ OPCs but not of late born OPCs [21]. Comparatively, in the developing spinal cord loss of *Ascl1* in progenitor cells that would normally produce neurons leads to a reduction in neurons and an increased expression of immature glial markers of both astrocyte and oligodendrocyte origin, but with no change in the OPC marker, Sox10 [22]. Consistently, *Ascl1* overexpression in the spinal cord has been shown to promote the maturation of OPCs into myelin forming oligodendrocytes [23]. Compounding the heterogeneity of the influence of Ascl1 on glial progenitors, it was recently shown in the spinal cord that Ascl1 affects both astrocytes and oligodendrocytes differentially in grey matter and white matter. In *Ascl1* knockouts, an increase in NFIA+, Olig2+, and Sox10+ glioblasts was observed in the grey matter, which is opposite to that observed in the white matter glial progenitor populations during later embryonic stages [24]. Interestingly, both *Neurog2* and *Ascl1* are expressed within progenitors within the tuberal hypothalamus but their role during hypothalamic gliogenesis has not yet been defined [25].

In this study we determined the spatiotemporal timing of gliogenesis in the tuberal hypothalamus by quantifying the timing and location of maturing oligodendrocyte, and to a lesser extent, astrocytes. We also employed *Ascl1*- and *Neurog2*-null mice to investigate the influence of these bHLH transcription factors in the progression of gliogenesis in this brain region. By characterizing the development of oligodendrocytes in this important brain region, we will be poised to better understand how disruption of gliogenesis might contribute to hypothalamic disease states, such as obesity.

## Methods

### Mouse strains and tissue preparation

Timed-pregnant wildtype CD1, *Neurog2* ^*GFP*KI^ [26, 27] and *Ascl1* ^*GFP*KI^ [26–28] were bred to obtain embryonic tissue samples. For embryonic staging, female mice were plug checked in the morning and those with a positive vaginal plug were assigned embryonic day (E) 0.5. For postnatal staging, the day of birth was assigned as postnatal day (P) 0. Genotyping was confirmed by embryonic tissue sampling using PCR with *Neurog2* ^*GFP*KI^ primers; mutant forward 5′-GGACATTCCCGGACACACAC-3′, mutant reverse 5′-GCATCACCTTCACCCTCTCC-3′, wildtype forward 5′-TAGACGCAGTGACTTCTGTGACCG-3′, wildtype reverse 5′-ACCTCCTCTTCCTCCTTCAACTCC-3′; and *Ascl1* ^*GFP*KI^ primers; mutant forward 5′-AACTTTCCTCCGGGGCTCGTTTC-3′, mutant reverse 5′-TGGCTGTTGTAGTTGTACTCCAGC-3′, wildtype forward 5′-TCCAACGACTTGAACTCTATGG-3′, wildtype reverse 5′-CCAGGACTCAATACGCAGGG-3′. Animal protocols were approved by the University of Calgary Animal Care Committee and follow the Guidelines of the Canadian Council of Animal Care.

For sample preparation, gravid females were anaesthetized with isoflurane and immediately decapitated. Embryos were removed and embryonic brains were extracted for E15.5 and E17.5 time points while whole embryonic heads were taken for E11.5 and E13.5 time points. For mutant samples, which have been outcrossed onto a CD1 background, CD1 wildtype samples were used as controls, with the exception of Fig. 7e, h where heterozygous *Ascl1* ^+/*GFP*KI^ animals were used as controls. For 5-Bromo-2′-deoxyuridine (BrdU) samples, 200 μl of 10 μg/μl BrdU was injected intraperitoneally into the pregnant dam. For neuronal birthdating studies, BrdU was injected at E11.5, E13.5 and E15.5 into the pregnant dam and resulting pups were sacrificed at P0. For proliferation studies, BrdU was injected at E13.5 and 30 min before decapitation of dam. Samples were fixed overnight with 4% paraformaldehyde in phosphate buffered saline (PBS), washed in PBS, then treated with 20% sucrose before being embedded in O.C.T. for cryosectioning.

### Immunofluorescence

Brain samples were cryosectioned at 10 μm with a selection sampling fraction of 1 for every 8 serial sections within our region of interest. Sections were treated with primary antibody overnight at 4 °C in 5% normal donkey or goat serum/PBS with 0.1% Tween-20 or Triton-X 100 followed by the appropriate fluorescently conjugated secondary antibody. Primary antibodies were as follows: Mouse anti-NeuN (Millipore; 1:400), Rat anti-BrdU (Cedar Lane; 1:300), Goat anti-Ki67 (Santa Cruz; 1:300), Rabbit anti-Ki67 (Abcam; 1:100), Goat anti-Sox9 (R&D systems; 1:40); Rabbit anti-Olig2 (Millipore; 1:500); Mouse anti-Olig2 (Millipore; 1:300), Goat anti-PdgfRα (R&D Systems; 1:150); Rat anti-SF-1 (graciously provided by Dr Taro Tachibana, Osaka City University JAPAN, 1:800); Rabbit anti-TTF-1 (alternatively Nkx2.1; Santa Cruz; 1:500), Goat anti-Sox10 (Santa Cruz, 1:500), Rabbit anti-pHH3 (Millipore; 1:500), Rabbit anti-Cyclin E (Santa Cruz; 1:200), Rabbit anti-Cyclin B1 (Santa Cruz; 1:200), Mouse anti-Cyclin D2 (ThermoFisher Scientific; 1:200), Rabbit anti-p57^kip^ (Sigma; 1:200), Rabbit anti-Aldh1L1 (Abcam; 1:500), Rat anti-MBP (Millipore; 1:50), and Rabbit anti-Cleaved Caspase 3 (Abcam; 1:800). All appropriate secondary antibodies were Donkey or Goat anti-IgG and Alexa Fluor conjugated (ThermoFisher Scientific; 1:200–1:400). All samples were counterstained with Hoechst nuclear stain (ThermoFisher Scientific; 1:1000).

### Quantification and statistical analysis

For cell number quantification, images were taken using a Ziess Axioplan 2 manual compound microscope with a Zeiss Axiocam HRc camera. Adobe Photoshop CS6 counting software was used to manually count individual and co-labeled cells. SF-1 staining-which marks the ventromedial hypothalamic nuclei-was used to denote the beginning and end of the tuberal hypothalamus across adjacent brain sections [29] and Nkx2.1 was used to verify the hypothalamic sulcus border at the dorsal edge of the tuberal hypothalamus. Cells were counted from 3 brain sections (descriptive counts) or 2 brain sections (mutant counts) in the rostral to mid tuberal hypothalamus for WT and mutant brains, respectively. Aldh1L1 cell counts were taken from the mid to caudal tuberal hypothalamus for control and mutant brains. Embryonic samples from more than one pregnant dam were used for each experimental group. Statistical differences between controls and mutants and between age time points were assessed using an ANOVA statistical test with Tukey post-hoc analysis or a Student’s *t*-*test* when applicable. Results are displayed as mean±standard deviation (SD).

## Results

### Glial progenitors first appear after E13.5 in the tuberal hypothalamus

**Fig. 1:**
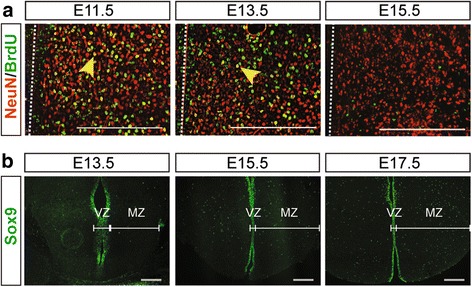
Progression of neurogenesis and gliogenesis in the tuberal hypothalamus of CD1 wildtype mice. **a** P0 brain sections of BrdU birthdating studies indicating the neurons, marked by NeuN, that were born at E11.5, E13.5 and E15.5 embryonic time points during neurogenesis in the tuberal hypothalamus. *Yellow* arrows indicate examples of NeuN+/BrdU+ co-labeled neurons, third ventricle location is highlighted with a *white* dotted line. **b** Sox9+ glioblasts in the tuberal hypothalamus at E13.5, E15.5 and E17.5. Scale bars equal 200 μm

Neurogenesis precedes gliogenesis throughout the CNS, prompting us to first ask when neurogenesis is complete in the tuberal hypothalamus, thereby providing a guideline as to when we would expect the onset of gliogenesis. Here we used BrdU to birthdate neurons born at various embryonic time points in the developing tuberal hypothalamus since terminally differentiated neurons become marked by the incorporation of BrdU during their final S-phase [30]. These birthdating experiments were performed by injecting BrdU into pregnant dams at E11.5, E13.5 and E15.5, and harvesting embryonic brains at P0. To define the rostrocaudal boarder of the tuberal hypothalamus, we immunolabeled adjacent sections with Steroidogenic factor 1 (SF-1, Nr5a1; Additional file 1: Figure S1), a definitive marker of the VMH [31–33] and whose rostrocaudal expression we had already determined [29]. Co-labeling of BrdU and NeuN, a pan-neuronal marker, revealed a large population of dual-labeled BrdU+/NeuN+ neurons (Fig. 1a; yellow cells) in P0 brains injected with BrdU at E11.5, which was diminished in P0 brains injected with BrdU at E13.5 and nearly absent in the P0 brains that were injected with BrdU at E15.5. Since the majority of cells at this latest time point had very little detectable BrdU incorporation, we postulated that E15.5 represents the end of the neurogenic window (Fig 1a). These data are consistent with previous reports [34], and lead us to choose E13.5 as our early time point as to when we might expect gliogenesis to commence, E15.5 as a period of active gliogenesis, and E17.5 to represent a period of oligodendrocyte maturation.

Next we asked when glioblasts first appeared in the tuberal hypothalamus by assaying Sox9 expression, a transcription factor required to specify a glial identity but that also labels neurogenic progenitors at the end of neurogenesis [35, 36]. At E11.5 and E13.5, Sox9 expression was mainly restricted to the ventricular zone (VZ) where multipotent neural progenitors are located, labeling the entire dorsal-ventral and rostral-caudal extent of the tuberal hypothalamic VZ (Additional file 1: Figure S2A and Fig. 1b). In contrast, by E15.5 and at E17.5, Sox9 expression was detected both in the VZ, representing a mix of neural and glial progenitors, and in the mantle zone (MZ; Fig. 1b), representing likely glial precursors, as the loss of ventricular contacts by dividing progenitors is a hallmark feature of glial precursors [37]. We thus conclude that the first glial precursors appear in the tuberal hypothalamus between E13.5 and E15.5.

### Olig2+ cells are restricted to a tight domain along the third ventricle in the tuberal hypothalamus

**Fig. 2:**
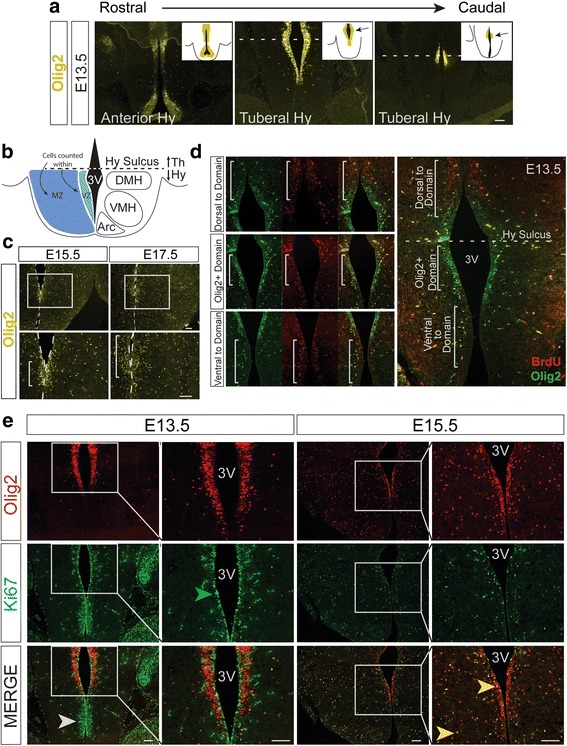
Olig2+ cells appear in a domain region along the 3rd ventricle in the tuberal hypothalamus. **a** Olig2+ cells located at the third ventricle (3 V) congregate in a domain in the tuberal area of the hypothalamus at the dorsal edge near the hypothalamic sulcus (*dotted white line*) at E13.5. Insets depict the Olig2 staining with the domain area indicated with arrows. **b** Illustration of coronal plane of nuclei of the tuberal hypothalamus to depict the hypothalamic sulcus dividing the thalamus (Th) from the hypothalamus (Hy). **c** Olig2+ cells at E15.5 and E17.5 with the Olig2+ domain area highlighted in the magnified image. *White* dotted line outlines the third ventricle. **d** BrdU and Olig2 co-immunostaining at E13.5 with areas dorsal to domain, the domain, and ventral to domain highlighted to outline differences in proliferative ability. **e** Ki67 and Olig2 co-immunostaining at E13.5 and E15.5. *White* arrow highlights pseudostratified proliferating cells ventral to domain, *green* arrow highlights a Ki67+ cell in domain area, *yellow* arrows indicate Ki67+/Olig2+ cells at the VZ and MZ. Scale bars equal 100 μm

To more accurately determine when glioblast differentiation commences in the tuberal hypothalamus, we examined the expression of Olig2, which marks a subset of glioblasts and maturing OPCs [15, 18]. At E13.5, Olig2+ cells lined the dorsoventral extent of the VZ surrounding the third ventricle in the anterior hypothalamus (Fig. 2a, left image), a region outside of the SF-1+ tuberal hypothalamic domain. In contrast, within the rostral area of the tuberal hypothalamus where SF-1 expression was first detected, Olig2+ cells begin to form a domain whereby they lined only a distinct portion of the VZ (Fig. 2a, middle image) that was located near the hypothalamic sulcus that separates the thalamus from the hypothalamus (Fig. 2a, b). More caudally within the SF-1+ tuberal hypothalamic area, Olig2+ cells become even further restricted to a smaller central domain of the VZ (Fig. 2a, right image), which also abuts the hypothalamic sulcus. This enrichment of Olig2+ cells in a central domain was most notable at E13.5, just prior to the release of glioblasts into the MZ (Fig. 2a), however some Olig2+ cells lining the ventricle of the tuberal hypothalamus were also observed starting at E11.5 (Additional file 1: Figure S2). By E15.5, when Olig2+ cells began to disperse away from the ventricle (Fig. 2c), this Olig2+ cluster was less robust and by E17.5 nearly unrecognizable (Fig. 2c). Olig2+ glioblasts thus occupy a restricted domain along the third ventricle within the early embryonic tuberal hypothalamus.

### Olig2+ progenitors along the VZ of the tuberal hypothalamus are not actively dividing

As progenitor cells lining ventricles are well known to be mitotically active [38], we next determined whether Olig2+ cells lining the third ventricle were mitotically active. To label rapidly proliferating S-phase cells, we injected BrdU at E13.5 and sacrificed the animals 30 min later. Interestingly, very few Olig2+/BrdU+ double labeled cells were observed within the Olig2+ domain along the tuberal hypothalamic VZ, whereas many BrdU+ cells were detected in the VZ directly dorsal and ventral to the Olig2+ enriched domain (Fig. 2d). In addition, Olig2+ cells that had migrated into the MZ were BrdU+, consistent with a proliferative glioblast fate (Fig. 2d).

**Fig. 3:**
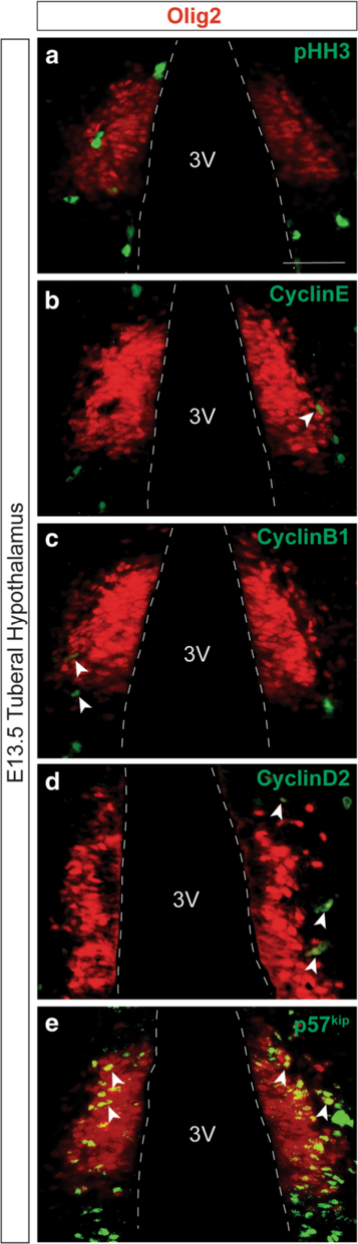
Olig2+ progenitors along the ventricular zone of the tuberal hypothalamus are not actively dividing. **a** Olig2+ cells located near the third ventricle (3 V) congregate in a domain in the tuberal area of the E13.5 hypothalamus that does not co-label with PPH3. **b**-**d** Olig2+ cells in the E13.5 tuberal hypothalamus show minimal co-labeling with the active cell cycle markers **b** Cyclin E, **c** Cyclin B1, and **d** Cyclin D2. **e** Olig2 and the cell cycle exit marker p57^kip^ show strong co-labeling in the E13.5 tuberal hypothalamus. *White* arrows highlight cells which co-labeled, while the *white* dotted line outlines the third ventricle. Scale bars equal 100 μm

To further test whether Olig2+ cells in the gliogenic domain were mitotically active, we co-labeled sections with Olig2 and the cell proliferation marker Ki67 (Fig. 2e). At E13.5, many Ki67+ cells were observed in the VZ ventral to the Olig2+ domain (Fig. 2e, white arrow), as was observed with BrdU+ immunostaining (Fig. 2d). Interestingly, the Ki67+ cells that were present within the restricted domain at E13.5 were directly adjacent to the ventricle wall and not across the ventricular zone (Fig. 2e, green arrow) and did not co-label with Olig2 (Fig. 2e, merge), further suggesting that Olig2+ cells within this VZ domain are not actively dividing at E13.5. Similarly, at E15.5, few Olig2+/Ki67+ cells were detected in the VZ, with the majority appearing in the MZ (Fig. 2e, yellow arrows). To further explore the cell cycle activity of Olig2+ cells within this domain, we co-labeled the E13.5 tuberal hypothalamus with Olig2 and the cell cycle markers phospho-histone H3 (pHH3), Cyclin E, Cyclin B1, and Cyclin D2 (Fig. 3a-d). We found no Olig2+/pHH3+ double-positive cells (Fig. 3a), and very few Olig2+/CyclinE+, Olig2+/CyclinB1+, and Olig2+/CyclinD2+ cells (Fig. 3b-d, white arrows). However, co-labeling with Olig2 and the cell cycle exit marker p57^kip^ [39] showed numerous double-positive cells (Fig. 3e, white arrows), consistent with our earlier findings that the majority of Olig2+ cells within the VZ are not actively dividing at E13.5. Taken together, these data suggest that Olig2 may have a cell cycle-restricted expression profile in the E13.5 hypothalamic VZ, largely being excluded from adjacent rapidly dividing progenitors.

### Changes in glial progenitor and precursor cell populations in the tuberal hypothalamus across development

**Fig. 4:**
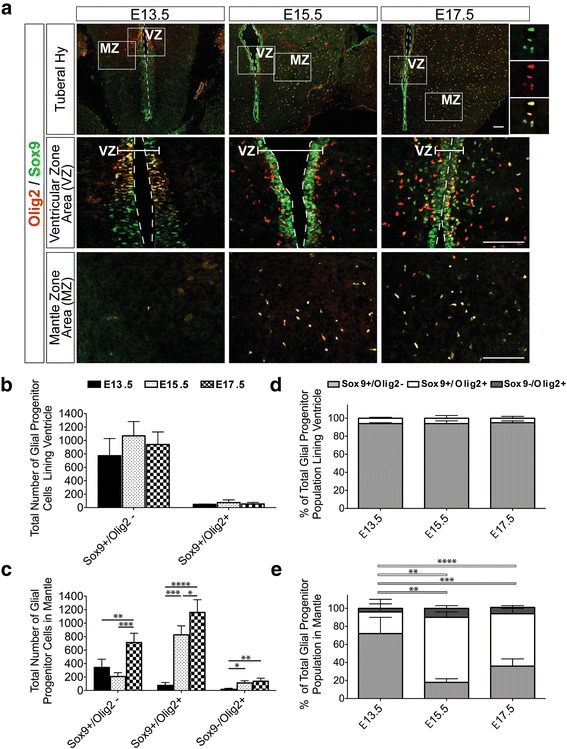
Glial progenitor cell populations in the developing embryonic tuberal hypothalamus at E13.5, E15.5 and E17.5. **a** Representative images of glioblast cells immunolabled with antibodies to Sox9 and Olig2 in the tuberal hypothalamic area of wildtype CD1 embryonic brains with boxed areas magnified for the ventricular zone (VZ; lateral area highlighted with bracket) and mantle zone (MZ). Insets show co-labeling of cells. 3rd ventricle outlined with dotted line. Glioblast cell counts of glioblast subpopulations that are (**b**) lining the ventricle in the VZ and **c** in the MZ. Proportion of glioblast subpopulations within the total glioblast population (**d**) lining the ventricle in the VZ and **e** in the MZ. Bar graphs represent mean ± SD (*n* = 4–5 embryos per group; 3 brain sections per embryo). Statistics; *P < 0.01, **P < 0.001, ***P < 0.0001, ****P < 0.00001. ANOVA with Tukey Post-Hoc. Scale bars equal 100 μm

To start to examine the differentiation of OPCs within the tuberal hypothalamus, we co-immunolabeled embryonic brain slices with Sox9 and Olig2. We first focused our analyses on cells in the VZ, examining the Olig2-enriched domain near the hypothalamic sulcus (Additional file 1: Figure S2A and Fig. 4a, VZ) and quantifying the Sox9+/Olig2- and Sox9+/Olig2+ cells. The number of Sox9+/Olig2- progenitor cells in the tuberal hypothalamic VZ did not significantly change across development (Fig. 4b): 773±256 cells at E13.5, 1069±211 cells at E15.5, and 940±186 cells at E17.5. Furthermore, the relative proportion of the Sox9+/Olig2- population along the VZ also did not change significantly over time: 94%±1% at E13.5, 94%±3% at E15.5, and 95%±2% at E17.5 (Fig. 4d). Similarly, the number of Sox9+/Olig2+ progenitor cells in the VZ, which were lower in number and likely glioblasts on their way to committing to an OPC fate, did not change significantly across embryonic time points (Fig. 4b): 48±5 cells at E13.5, 75±40 cells at E15.5, and 54±24 cells at E17.5. And consistently, the relative proportion of Sox9+/Olig2+ in the VZ also did not fluctuate across development: 6%±1% at E13.5, 6%±1% at E15.5 and 5%±2% at E17.5 (Fig. 4d). Combined, at the VZ neither Sox9+/Olig2- nor Sox9+/Olig2+ total cell counts or relative proportions were significantly different across time points (Fig. 4b, d). We did not detect any Sox9-/Olig2+ cells at the ventricle at any time point starting at E11.5, which is when Sox9 is just beginning to be expressed in the tuberal hypothalamus (Additional file 1: Figure S2A and data not shown). The tuberal hypothalamic VZ thus has a stable pool of progenitors expressing glial markers at mid-to-late embryonic time points.

Given that glial precursors migrate away from the VZ and into the MZ where they proliferate and mature, we next examined the number of progenitors expressing Sox9 and/or Olig2 in the MZ, observing all three possible populations of glioblasts, namely Sox9+/Olig2-, Sox9+/Olig2+, Sox9-/Olig2+ (Fig. 4c, e). We first examined Sox9+/Olig2+ cells, considered to be early-stage glioblasts and OPCs, and found a significant and dramatic increase in number of these cells from E13.5 to E15.5 and a further increase at E17.5: 76±43 cells at E13.5, 826±134 cells at E15.5, and 1159±188 cells at E17.5 (Fig. 4c). Moreover, we also quantified the relative proportion of the Sox9+/Olig2+ population within the total Sox9+ and Olig2+ glioblast populations in the MZ and found Sox9+/Olig2+ population likewise increased from E13.5 to E15.5 and then remained constant to E17.5: 24%±14% at E13.5, 72%±6% at E15.5, and 58%±6% at E17.5 (Fig. 4e). We next examined Sox9+/Olig2- cells in the MZ and found this population to be relative low and maintained from E13.5 to E15.5 with a significant increase at E17.5: 342±123 cells at E13.5, 206±cells at E15.5, and 711±138 cells at E17.5 (Fig. 4c). This was in contrast to the relative proportion of Sox9+/Olig2- glioblasts within the total Sox9+ and Olig2+ populations, which decreased significantly from E13.5 to E15.5 but remained constant to E17.5: 72%±18% at E13.5, 18%±4% at E15.5, and 36%±8% at E17.5 (Fig. 4e). Finally, we quantified the population of Sox9-/Olig2+ cells, which are likely maturing OPCs (see next section). Unlike in the VZ where no Sox9-/Olig2+ cells were detected from E13.5 to E17.5, within the MZ we identified a distinct population that significantly increased from E13.5 to E15.5 and remained constant thereafter to E17.5: 17±14 cells at E13.5, 111±32 cells at E15.5, and 138±44 cells at E17.5 (Fig. 4c). In contrast, the relative proportion of Sox9-/Olig2+ glioblasts remained constant across E13.5, E15.5 and E17.5: 4%±5% at E13.5, 10%±3% at E15.5, and 7%±2% at E17.5 (Fig. 4e). Taken together, these data demonstrate a major expansion in the MZ from E13.5 to E15.5 of the Sox9+/Olig2+ population, thought to be OPCs, as well as a pool of Sox9-/Olig2+ cells that may correspond to differentiating OPCs. We also observe a second wave of later expansion of the Sox9+/Olig2- population, thought to be early-stage glioblasts and/or astrocyte precursors between E15.5 and E17.5.

### Oligodendrocyte progenitor and precursor cell populations in the tuberal hypothalamus

**Fig. 5:**
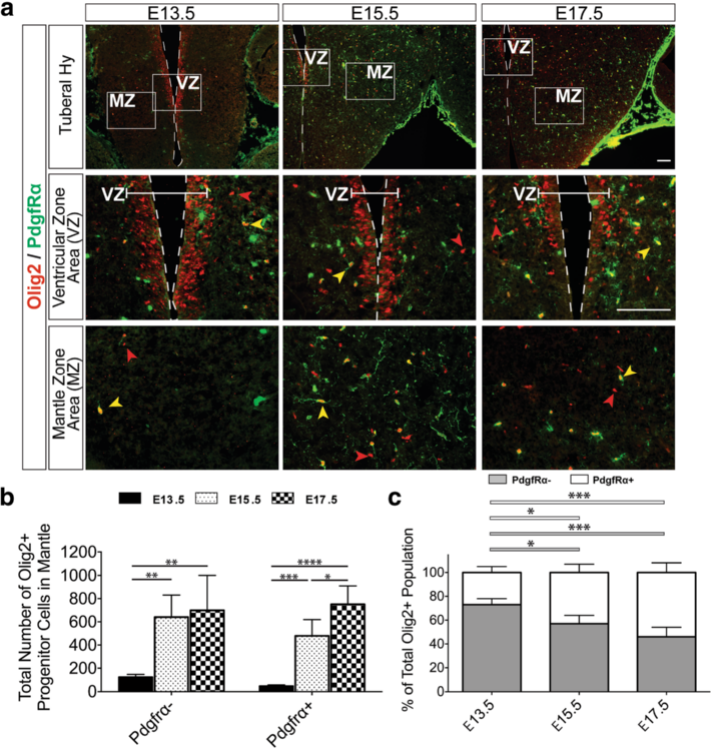
Olig2+ oligodendrocyte progenitor cell populations in the developing embryonic tuberal hypothalamus at E13.5, E15.5 and E17.5. **a** Representative images immunolabled with antibodies to PdgfRα and Olig2 in the tuberal hypothalamic area of wildtype CD1 embryonic brains, with boxed areas magnified for the ventricular zone (VZ; area highlighted with bracket) and mantle zone (MZ). *Red* arrows indicate examples of Olig2+/PdgfRα- cells, *green* arrows indicate examples of Olig2+/PdgfRα + cells. Third ventricle outlined with dotted line. **b** Cell counts of Olig2+ and PdgfRα + (OPCs) or PdgfRα- (glioblasts) populations in the MZ. **c** Proportion of PdgfRα + co-labeled and PdgfRα- cell populations within the total Olig2+ population in the MZ. Bar graphs represent mean ± SD (*n* = 4–5 embryos per group; 3 brain sections per embryo). Statistics; *P < 0.01, **P < 0.001, ***P < 0.0001, ****P < 0.0001. ANOVA with Tukey Post-Hoc. Scale bars equal 100 μm

Although the majority of Olig2+ cells go on to become OPCs, a portion of these cells can give rise to astrocytes [15, 19], so next we examined the population of Olig2+ glioblasts committed to becoming OPCs across development. We employed the OPC marker PdgfRα, and quantified the number of Olig2+ cells that were either PdgfRα- or PdgfRα+. At E11.5 we did not detect PdgfRα + cells in the developing hypothalamus (Additional file 1: Figure S2B), consistent with oligodendrogenesis occurring after neurogenesis and just prior to E13.5. Moreover, from E13.5 to E17.5 no PdgfRα + cells were identified within the VZ (Fig. 5a, VZ) and all Olig2+/PdgfRα+ cells were localized in the MZ, consistent with their expression in differentiating OPCs and oligodendrocytes (Fig. 5a). Although PdgfRα labels the cell body and processes of OPCs and Olig2 is an OPC nuclear marker, dual-labeling demonstrated quantifiable overlapping expression in Olig2+/PdgfRα+ cells (Fig. 5a, yellow arrows) that were distinguishable from Olig2+/PdgfRα- cells (Fig. 5a, red arrows). Quantification of Olig2+/PdgfRα- cells, the majority of which will become committed OPCs at these later embryonic time points [15], significantly increased from E13.5 to E15.5 and remained relatively constant at E17.5, consistent with our previous findings in Fig. 4: 122±23 cells at E13.5, 641±189 cells at E15.5, and 699±301 cells at E17.5 (Fig. 5b). In contrast, the population of Olig2+/PdgfRα+ cells, which are considered committed to an OPC fate and undergoing differentiation, significantly increased from E13.5 to E15.5 and continued to increase at E17.5: 44±12 cells at E13.5, 479±140 cells at E15.5, and 751±158 cells at E17.5 (Fig. 5b). The relative proportion of Olig2+/PdgfRα+ cells in the total Olig2+ population significantly increased from E13.5 to E15.5 but remained relatively constant thereafter to E17.5, demonstrating that the committed OPC population is about half of the total Olig2+ cell population at E15.5 and onwards: 27%±5% at E13.5, 43%±7% at E15.5, and 54%±8% at E17.5 (Fig. 5c). Combined, these data reveal a significant expansion of both Olig2+ glioblasts and Olig2+/PdgfRα+ differentiating OPCs/oligodendrocytes from E13.5 to E15.5, with maturing cells increasing further from E15.5 to E17.5.

### Maturing oligodendrocyte and astrocyte populations in the developing tuberal hypothalamus

**Fig. 6:**
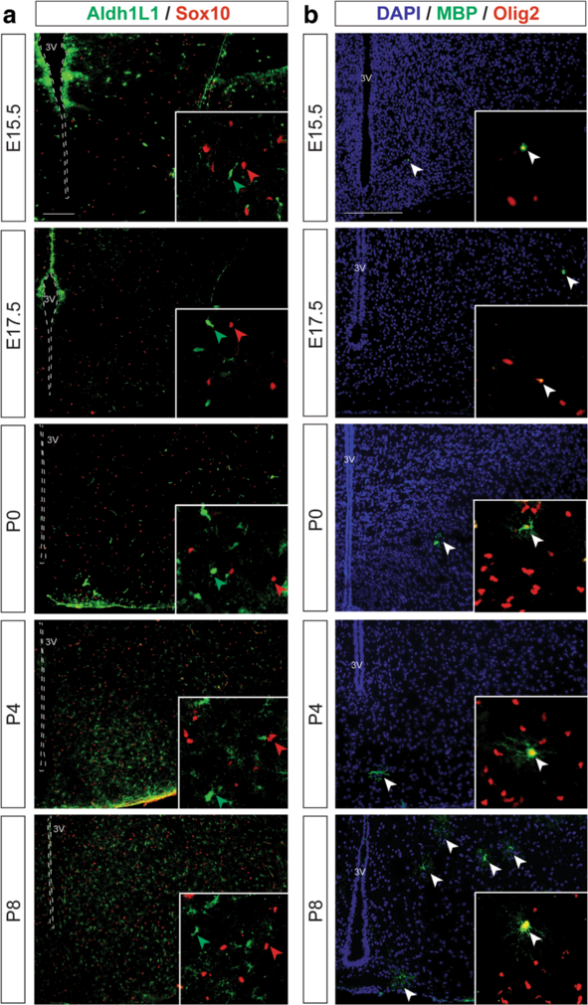
Maturing oligodendrocyte and astrocyte populations in the developing tuberal hypothalamus. **a** Representative images of astrocytes in the E15.5, E17.5, P0, P4 and P8 tuberal hypothalamic area of wildtype CD1 embryonic brains immunolabled with antibodies to Aldh1L1 and Sox10, with insets showing higher magnification to confirm there is no co-labeling of Aldh1L1+ astrocytes and Sox10+ cells. **b** Representative images of premyelinating and myelinating oligodendrocytes in the E15.5, E17.5, P0, P4 and P8 tuberal hypothalamic area of wildtype CD1 embryonic brains immunolabled with antibodies to Olig2 and MBP, with insets showing higher magnification to confirm there is co-labeling of Olig2+ cells with MBP. 3rd ventricle outlined with dotted line. Scale bars equal 250 μm

As previously mentioned, the majority of Olig2+ cells go on to become OPCs, however a portion of these cells can give rise to astrocytes [15, 19]. Therefore, we next examined astrocyte development in the tuberal hypothalamus using Aldehyde dehydrogenase 1 (Aldh1L1) [40], one of the few astrocyte markers expressed embryonically. We compared Aldh1L1 expression with that of Sox10, which definitely labels maturing OPCs given its role as a key determinant in terminal oligodendrocyte differentiation, survival, and migration [11, 41]. Since no Aldh1L1+ cells were identified prior to E15.5 (data not shown), we co-labeled E15.5 to P8 hypothalamic sections with Aldh1L1 and Sox10 and only singly positive Aldh1L1+ cells (Fig. 6a, green arrows) or Sox10+ cells (Fig. 6a, red arrows) were identified, consistent with Sox10 being expressed specifically in oligodendrocyte lineages. Despite Aldh1L1+ astrocytes being detected as early as E15.5 and E17.5 (Fig. 6a), we only began to observe astrocyte branching and maturation at P0, which increased significantly from P4 to P8 (Fig. 6a). We also observed an increase in the overall number of astrocytes expressing Aldh1L1 from E15.5 to P8 (Fig. 6a), suggesting that astrocytogenesis is occurring alongside, although slightly delayed from, oligodendrogenesis. We next examined the population of Olig2+ OPCs that expressed the maturing oligodendrocyte marker Myelin basic protein (MBP), which labels both premyelinating and myelinating oligodendrocytes [40]. Across development in the tuberal hypothalamus, we were able to detect MBP as early as E15.5 and E17.5 (Fig. 6b, white arrows); however, we only observed oligodendrocyte branching from P0 to P8 (Fig. 6b, white arrows). We also observed an increase in the number of maturing oligodendrocytes expressing MBP from E15.5 to P8 (Fig. 6b, white arrows), and at all time points the MBP+ cells also co-labeled with Olig2 (Fig. 6b inset, white arrows). Together, these data demonstrate that Olig2+ cells can go on to become mature oligodendrocytes that myelinate their axons. Furthermore, although a small population of Olig2+ cells can give rise to astrocytes that co-label with Aldh1L1 (see next section), Sox10 specifically marks the oligodendrocyte lineage and can thus be used to distinguish Olig2+ glioblasts that will become oligodendrocytes (e.g., Olig2+/Sox10+) away from Olig2+ glioblasts that will become astrocytes (e.g., Olig2+/Sox10-).

### Altered glial progenitor and precursor cell populations in *Ascl1* and *Neurog2* mutant embryos

**Fig. 7:**
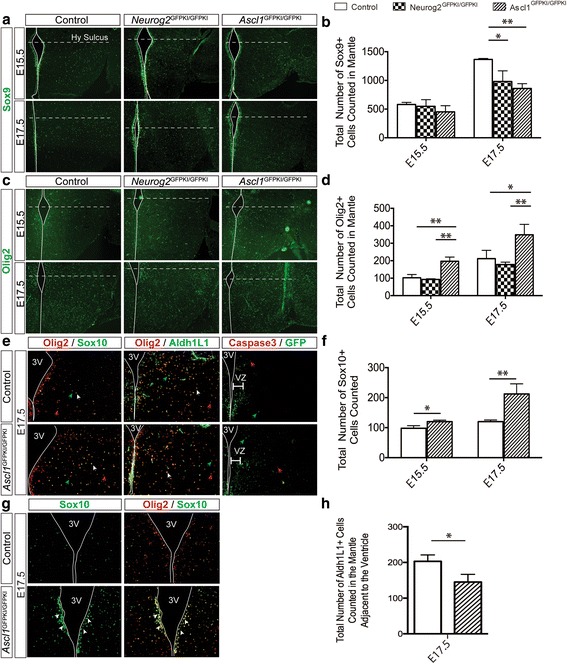
Mid- to late- embryonic Sox9+, Olig2+, Sox10+ glial populations in the tuberal hypothalamus of Control, Neurog2 ^GFPKI/GFPKI^ and Ascl1 ^GFPKI/GFPKI^ mutant embryo brains. Example images of (**a**) Sox9+, **c** Olig2+ glial precursors at E15.5 and E17.5. Total number of (**b**) Sox9+ and **d** Olig2+ glioblasts counted in the mantle region in control, Neurog2 ^GFPKI/GFPKI^ and Ascl1 ^GFPKI/GFPKI^ embryo brains. **e** Olig2/Sox10, Olig2/Aldh1L1 and cleaved Caspase3/GFP immunolabeled E17.5 control and Ascl1 ^GFPKI/GFPKI^ mutant embryo brains. Total number of (**f**) Sox10+ OPCs and **g** Aldh1L1+ astrocytes counted in control and Ascl1 ^GFPKI/GFPKI^ embryo brains. **h** Sox10 and Sox10/Olig2 immunolabeled E17.5 control and Ascl1 ^GFPKI/GFPKI^ mutant embryo brains. 3 V outlined with solid *white* line, hypothalamic sulcus indicated with dotted *white* line. Bar graphs represent mean ± SD (*n* = 3 embryos per group; 2 brain sections per embryo). Statistics: *P < 0.01, **P < 0.001, ***P < 0.0001. ANOVA with Tukey Post-Hoc or Student’s *t*-test

We next investigated whether *Neurog2* and/or *Ascl1* were required for gliogenesis in the developing embryonic tuberal hypothalamus by using the *Neurog2* ^*GFP*KI/*GFP*KI^ and *Ascl1* ^*GFP*KI/*GFP*KI^ mutant mice whereby GFP replaces the coding regions of *Neurog2* and *Ascl1*, respectively, thereby creating null alleles [26–28]. The proneural gene *Ascl1* was a strong candidate for playing a role during gliogenesis given its broad expression within progenitors across the VZ during embryonic tuberal hypothalamic development (Additional file 1: Figure S3; E12.5 and E14.5), and overlapping expression with the glioblast and OPC markers Sox9 (Fig. 1b) and Olig2 (Fig. 2). Since the major expansion of the OPC population is observed from E15.5 to E17.5 in the developing tuberal hypothalamus, we restricted our studies to this time period. First, we quantified whether there was a change in Sox9+ glioblasts in the MZ of mutant hypothalami (Fig. 7a, b). We found that the number of Sox9+ cells within the MZ was not significantly different at E15.5 between *Neurog2* ^*GFP*KI/*GFP*KI^ mutants (548±116 cells), *Ascl1* ^*GFP*KI/*GFP*KI^ mutants (451±108; Fig. 7b) and wild-type controls (580±36 cells). In contrast, at E17.5, an approximately 1.5-fold significant decrease in Sox9+ cells was detected in both the Neurog2^*GFP*KI/*GFP*KI^ mutants (980±187 cells) and the Ascl1^*GFP*KI/*GFP*KI^ mutants (859±83 cells) relative to wild-type controls (1365±17; Fig. 7b). Moreover, we did not observe any changes in apoptosis in Ascl1^*GFP*KI/*GFP*KI^ mutants by staining for cleaved Caspase3 (Fig. 7e).

To resolve whether the decrease in Sox9+ cells in the developing tuberal hypothalamus in *Neurog2* ^*GFP*KI/*GFP*KI^ and *Ascl1* ^*GFP*KI/*GFP*KI^ mutants affected oligodendrocyte development, we quantified changes in Olig2, a general marker for glioblasts and OPCs, in the MZ (Fig. 7c, d). In the MZ there was an almost 2-fold increase in Olig2+ cells in *Ascl1* ^*GFP*KI/*GFP*KI^ mutants at E15.5 (197±24 cells) that was also observed at E17.5 (349±60 cells; Fig. 7d), relative to controls at E15.5 (102±19 cells) and E17.5 (212±47 cells). In contrast, no difference was observed in Olig2+ cells in *Neurog2* ^GFPKI/GFPKI^ mutants relative to controls at E15.5 (93±4 cells) and E17.5 (178±14; Fig. 7d).

Given that a percentage of Olig2+ cells can become astrocytes, we asked if the increase in the Olig2+ population in Ascl1^*GFP*KI/*GFP*KI^ mutants was giving rise to an oligodendrocyte or astrocyte lineage. To do so, we performed immunohistochemistry with Sox10 (Fig. 7e, f) and showed a statistical increase in Sox10+ cells at both E15.5 (120±6 cells) and E17.5 (212±34 cells) in the Ascl1 ^*GFP*KI/*GFP*KI^ mutant relative to controls at E15.5 (98±9 cells) and at E17.5 (119±6 cells; Fig. 7f). Although an increase in Sox10+ cells in the Ascl1^*GFP*KI/*GFP*KI^ mutant (Fig. 7f) suggested an increase in the oligodendrocyte lineage, we also examined astrocytes in Ascl1^*GFP*KI/*GFP*KI^ mutants using Aldh1L1 to mark astrocytes. Co-labeling with Olig2 and Aldh1L1 demonstrated a decrease in the Aldh1L1+ astrocyte population in Ascl1^*GFP*KI/*GFP*KI^ mutants (145±12 cells) as compared to controls (203±10 cells; Fig. 7g). The decrease in astrocytes was found in the MZ just adjacent to the VZ where Olig2+ glioblasts are migrating outward (Fig. 7e, data not shown), thereby supporting a role for Ascl1 in directing oligodendrocyte versus astrocyte fates. It is interesting to note that these changes also coincided with the appearance of Sox10+ cells in the VZ in Ascl1^*GFP*KI/*GFP*KI^ mutants (Fig. 7h, arrows), which were also found to be Olig2+ (Fig. 7h). Together, these data suggest that *Ascl1* but not *Neurog2* plays a key role in late embryonic oligodendrogenesis in the developing tuberal hypothalamus.

## Discussion

### Spatiotemporal progression of gliogenesis in the embryonic tuberal hypothalamus

**Fig. 8:**
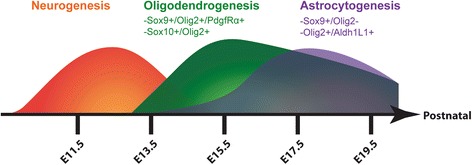
Progression of timing of neurogenesis, oligodendrogenesis and astrocytogenesis in the embryonic tuberal hypothalamus. Following a first wave of neurogenesis which ends around E14.5, gliogenesis begins with a second wave producing oligodendrocyte progenitors and OPCs with a peak in population production around E15.5. Subsequently a third wave of astrocytogenesis produces astrocyte precursor cells between E15.5 and E17.5

Here we show the progression of gliogenic expansion in the embryonic tuberal hypothalamus. We found that both Sox9+ and Olig2+ glial progenitors form along the third ventricle and migrate out into the MZ starting around E13.5, a time point consistent with post-neurogenesis. Sox9+/Olig2+ populations, which are likely early-stage OPCs, expand their population substantially from E13.5 to E15.5 with another, smaller, expansion onwards to E17.5. This expansion is further demonstrated by the increase in the committed OPC population, Olig2+/PdgfRα+, from E13.5 to E15.5 and continuing to E17.5. There is also an Olig2+/PdgfRα- glioblast population that mirrors this growth from E13.5 to E15.5, which is perhaps a committed astrocytic lineage. Combined, we propose that the major expansion of oligodendrocyte progenitors and precursors occurs from E13.5 to E15.5 when the first gliogenic wave in the tuberal hypothalamus produces OPCs (Fig. 8).

We report that Olig2+ cells are restricted to a dorsal domain adjacent to the third ventricle near the hypothalamic sulcus of the tuberal hypothalamus, and propose that OPCs are formed and migrate out from this domain to establish the MZ glioblasts, which then proliferate to generate the expansion of oligodendroctyes. Previous studies have described similar OPC-producing domains in other CNS regions, as nicely reviewed by Nicolay et al. [7]. Moreover, this Olig2+ domain demonstrates a reduced proliferative capacity at E13.5 in comparison to the surrounding ventricle progenitors that appear as a pseudo-stratified layer of Ki67+ and BrdU+ cells likely in various stages of an active cell cycle due to the appearance of interkinetic nuclear migration [42]. Interestingly, we did observe a small population of Ki67+ cells within the Olig2+ domain but they were largely Olig2- in expression and were tightly localized to the ventricular wall and did not assume a pseudo-stratified organization. Here we propose two possible populations of progenitor cells might co-exist within this Olig2+ domain region, which may have distinctive purposes. Firstly, the Ki67+/Olig2- progenitor cells directly adjacent to the ventricle (Fig. 2e) may be in the process of slowing their cell cycle, inferred by their lack of BrdU accumulation but positive expression of Ki67, and ultimately becoming an alternative neural progenitor type. In fact, it was recently discovered that adult stem cells are derived from a subset of the rapidly dividing neural progenitor pool of cells that have slowed their cell cycle starting between E13.5 and E15.5 in order to elongate their cycling potential into adulthood [43]. Coincidentally, overlapping this region of the Olig2+ domain in the tuberal hypothalamic VZ are also progenitors that later generate α-tanycytes, which are known to have adult stem cell properties [44, 45], raising the exciting notion that these Ki67+/Olig2- progenitor cells are being reserved for tanycytic adult neurogenesis.

Secondly, we also propose that the Ki67-/Olig2+ progenitor population at the VZ are slowing their cell cycle to become specified as glioblasts, and have paused their mitotic activity in anticipation of movement as they prepare to exit the VZ. Consistent with this idea, we observe accumulation of the cell cycle arrest marker, p57^kip^, in cells at the outer edge of the VZ that co-label with Olig2 (Fig. 3e). This notion would be consistent with our findings here that Olig2+ cells expand significantly out in the MZ after E13.5 and acquire a highly proliferative state in the mantle. Future studies are needed to fully understand the proliferative capacity of cells within this Olig2+ domain, although here we demonstrate that at least two distinct populations reside within this dorsal VZ domain.

### Ascl1 influences gliogenesis in the tuberal hypothalamus

Here we uncovered a unique role for Ascl1 in gliogenesis in the tuberal hypothalamus. Both *Neurog2*- and *Ascl1*-null mutants showed a marked reduction in E17.5 Sox9+ cells, although only the *Ascl1* single mutant showed an increase in the Olig2+ and Sox10+ cells at both at E15.5 and at E17.5 and a decrease in astrocytes at E17.5, suggesting that oligodendrocyte populations are increased in the *Ascl1* mutant. Since Sox9 is co-expressed with Olig2 in early OPC populations, this increase in Olig2+ and Sox10+ cells and concomitant decrease in the Sox9+ is likely due to a precipitous increase in OPC maturation in the *Ascl1* mutant background. These changes also coincide with the appearance of Sox10+ cells in the VZ in Ascl1^*GFP*KI/*GFP*KI^ mutants, which were also found to be Olig2+. This suggests that Ascl1 is required to repress precocious OPC development and oligodendrocyte lineage commitment of Olig2+ cells lining the ventricle [46–48]. This is further supported by the overlap of Ascl1 and Olig2 expression embryonically in progenitors lining the ventricle. However, it is important to note that given immature glia cells divide during their migratory routes, and the number of divisions determine the number of mature glia cells, it is also possible that in the absence of Ascl1 astrocytes do not divide to the same extent. Interestingly, these findings in the tuberal hypothalamus are in direct contrast to those in the cerebellum whereby the *Ascl1* loss-of-function mutants were shown to have a reduction in Olig2+ cells and an increase in Sox9+ cells at E18.5 [16]. Furthermore, a study by Parras et al. using *Ascl1*-null mice in the telencephalon demonstrate a decrease in PdgfRα+ OPCs during early gliogenesis at E13.5 that later resolved by E17.5. The OPC population that was restored late embryonically in their study was suggested to be due to either an increase in the rate of proliferation of OPCs or, more likely, an increase in specification of OPCs from uncommitted progenitors that reflect a compensatory mechanism for OPC specification in the absence of *Ascl1* [21]. Here we only examined the mid- (E15.5) to late- (E17.5) stages of embryonic gliogenesis, and thus, it is possible we missed an initial decrease in OPCs earlier (e.g., E13.5), making it possible that we misinterpreted what was ultimately simply overcompensation at later stages. However, given that we show very few PdgfRα OPCs in the developing tuberal hypothalamus at E13.5, we believe it unlikely that we missed an OPC phenotype earlier than E15.5. Moreover, in agreement with our findings here, reports in the spinal cord also using an *Ascl1* knockout show an increase in Olig2+ cells at E17.5, which the authors attribute to aberrant proliferation as Ki67 and BrdU were also increased [22], and are further supported by a more recent report in the spinal cord showing an increase in Olig2+ and Sox10+ cells at E18.5 in grey matter oligodendrocytes [24]. Taken together, our data are consistent with a role for *Ascl1* in oligodendrogenesis, and suggests that whether *Ascl1* is required or represses an OPC fate might be CNS region-specific.

Mechanistically, we propose that with *Ascl1* eliminated, the competency of progenitor cells to maintain a proliferative state may be reduced since oscillatory Ascl1 cannot cross-repress the other bHLH factors that are required to maintain a true progenitor state. Indeed, Ascl1 and Neurog2 are both key components of a progenitor transcription factor oscillatory process whereby progenitors continue to divide instead of exiting the cell cycle [46] because key transcription factors are expressed for short periods to cross-repress other transcription factors in an oscillatory manner. A lineage becomes committed once a transcription factor stops oscillating and experiences sustained expression [47]. Specifically, when considering the oscillatory activity of Hes1, Ascl1 and Olig2 in neural progenitor cells, Ascl1 oscillations are required to maintain a proliferating neural progenitor state, while sustained Ascl1 expression promotes a neuronal fate. Alternatively, a decrease in expression of Ascl1 with a sustained expression of Olig2 or Hes1 promotes an oligodendrocyte and astrocyte fate, respectively [47, 48]. Thus, in the tuberal hypothalamus, we postulate that the elimination of *Ascl1* in Olig2+ progenitor cells restricts the neuronal fate potential of these cycling cells, thereby inducing these progenitors to initiate precocious OPC development.

## Conclusions

In summary, this study provides insight into the spatiotemporal timing of gliogenesis in the tuberal hypothalamus revealing that peak production of developing oligodendrocytes occurs between E15.5 and E17.5. We also show a role for Ascl1 in this region that is more consistent with Ascl1 requirements in the developing spinal cord than in the cortex and cerebellum.

## Additional file

Additional file 1:
**Figure S1:** Examples of coronal expression pattern of SF-1, a neuronal VMH marker, used to indicate the rostral-caudal borders of the tuberal hypothalamus at E11.5, E13.5, E15.5, E17.5 and P0. VMH is outlined with dotted white lines. **Figure S2:** E11.5 tuberal hypothalamus immunolabeled with (A) Olig2/Sox9 and (B) Olig2/Pdgfrα 3rd ventricle (3 V) is outlined with dotted white lines. VZ indicates the ventricular zone and MZ indicates the mantle zone. **Figure S3:** *Ascl1* expression location in the *Ascl1* ^*GFPKI/+*^ mouse in the ventral to mid 3 V in the tuberal hypothalamus at E12.5 and E14.5. Top panels present mouse in the ventral to mid 3 V in the tuberal hypothalamus at E12.5 and E14.5. Top panels present in situ hybridization showing *Ascl1* mRNA expression and bottom panels present corresponding GFP tracing. (PDF 13568 kb)

## Abbreviations

ARC: Arcuate nucleus
Ascl1: Achaete-scute homolog
bHLH: Basic helix-loop-helix family
BrdU: 5-Bromo-2′-deoxyuridine
CNS: Central nervous system
DMH: Dorsomedial hypothalamus
GFP: Green fluorescent protein
MZ: Mantle zone
Neurog2: Neurogenin 2
OPC: Oligodendrocyte precursor cell
PBS: Phosphate buffered saline
SD: Standard deviation
SF-1: Steroidogenic factor 1
VMH: Ventromedial hypothalamus
VZ: Ventricular zone
