# Supplementary material for: Oligodendrocyte development in the embryonic tuberal hypothalamus and the influence of Ascl1
Source: Neural Dev. 2016 Nov 18;11:20. doi: 10.1186/s13064-016-0075-9 (PMC5116181; doi:10.1186/s13064-016-0075-9)
Supplement: Additional file 1: — Figure S1: Examples of coronal expression pattern of SF-1, a neuronal VMH marker, used to indicate the rostral-caudal borders of the tuberal hypothalamus at E11.5, E13.5, E15.5, E17.5 and P0. VMH is outlined with dotted white lines. Figure S2: E11.5 tuberal hypothalamus immunolabeled with (A) Olig2/Sox9 and (B) Olig2/Pdgfrα 3rd ventricle (3 V) is outlined with dotted white lines. VZ indicates the ventricular zone and MZ indicates the mantle zone. Figure S3: Ascl1 expression location in the Ascl1 GFPKI/+ mouse in the ventral to mid 3 V in the tuberal hypothalamus at E12.5 and E14.5. Top panels present mouse in the ventral to mid 3 V in the tuberal hypothalamus at E12.5 and E14.5. Top panels present in situ hybridization showing Ascl1 mRNA expression and bottom panels present corresponding GFP tracing. (PDF 13568 kb) [file 13064_2016_75_MOESM1_ESM.pdf]

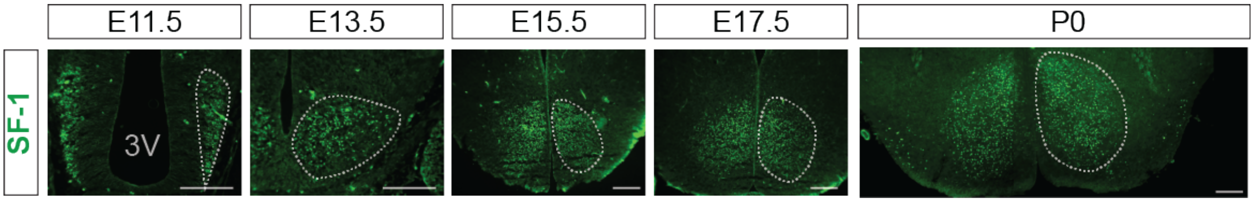

Supplementary Figure 1: Examples of coronal expression pattern of SF-1, a neuronal VMH marker, used to indicate the rostral-caudal borders of the tuberal hypothalamus at E11.5, E13.5, E15.5, E17.5 and P0. VMH is outlined with dotted white lines. P03VE11.5E13.5E15.5E17.5SF-1

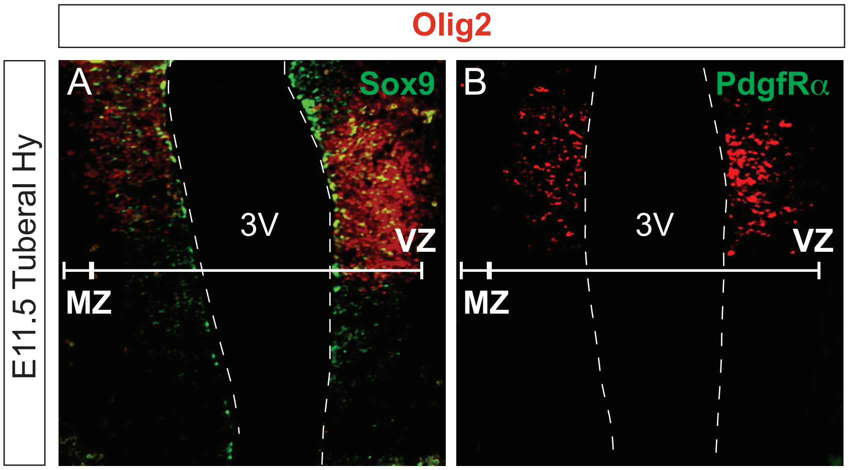

Supplementary Figure 2: E11.5 tuberal hypothalamus immunolabeled with (A) Olig2/Sox9 and (B) Olig2/PdgfRα. 3rd ventricle (3V) is outlined with dotted white lines. VZ indicates the ventricular zone and MZ indicates the mantle zone.

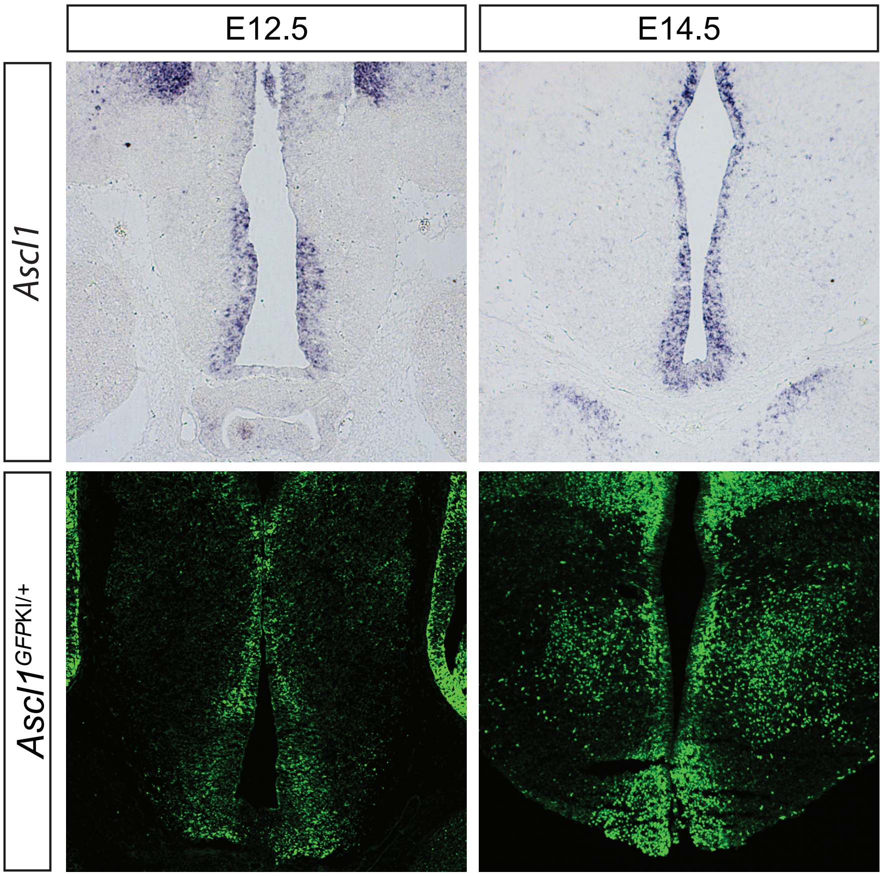

Supplementary Figure 3. Expression of Ascl1 in E12.5 and E14.5 in the developing tuberal hypothalamus. Top panel: in situ hybridization using *Ascl1* riboprobe. At E12.5, Ascl1 expression was observed in the VZ ventral to the hypothalamic sulcus. By E14.5, Ascl1 expression had expanded to progenitors throughout the VZ, including dorsal to the hypothalamic sulcus. Bottom panel: GFP expression in $Ascl1^{GFPKI}$ animals. At E12.5, GFP expression was observed in progenitors throughout the VZ. By E14.5, GFP+ cells were observed in the VZ and also in the MZ, likely due to a short-term lineage trace of Ascl1+ progenitors.
